# Butterfly Wings Are Three-Dimensional: Pupal Cuticle Focal Spots and Their Associated Structures in *Junonia* Butterflies

**DOI:** 10.1371/journal.pone.0146348

**Published:** 2016-01-05

**Authors:** Wataru Taira, Joji M. Otaki

**Affiliations:** The BCPH Unit of Molecular Physiology, Department of Chemistry, Biology and Marine Science, Faculty of Science, University of the Ryukyus, Nishihara, Okinawa 903-0213, Japan; Technische Universität Dresden, GERMANY

## Abstract

Butterfly wing color patterns often contain eyespots, which are developmentally determined at the late larval and early pupal stages by organizing activities of focal cells that can later form eyespot foci. In the pupal stage, the focal position of a future eyespot is often marked by a focal spot, one of the pupal cuticle spots, on the pupal surface. Here, we examined the possible relationships of the pupal focal spots with the underneath pupal wing tissues and with the adult wing eyespots using *Junonia* butterflies. Large pupal focal spots were found in two species with large adult eyespots, *J*. *orithya* and *J*. *almana*, whereas only small pupal focal spots were found in a species with small adult eyespots, *J*. *hedonia*. The size of five pupal focal spots on a single wing was correlated with the size of the corresponding adult eyespots in *J*. *orithya*. A pupal focal spot was a three-dimensional bulge of cuticle surface, and the underside of the major pupal focal spot exhibited a hollowed cuticle in a pupal case. Cross sections of a pupal wing revealed that the cuticle layer shows a curvature at a focal spot, and a positional correlation was observed between the cuticle layer thickness and its corresponding cell layer thickness. Adult major eyespots of *J*. *orithya* and *J*. *almana* exhibited surface elevations and depressions that approximately correspond to the coloration within an eyespot. Our results suggest that a pupal focal spot is produced by the organizing activity of focal cells underneath the focal spot. Probably because the focal cell layer immediately underneath a focal spot is thicker than that of its surrounding areas, eyespots of adult butterfly wings are three-dimensionally constructed. The color-height relationship in adult eyespots might have an implication in the developmental signaling for determining the eyespot color patterns.

## Introduction

Butterfly wing color patterns are highly diverse, but it has been thought that they mostly derive from the nymphalid ground plan [1–5]. The nymphalid ground plan is composed of three major symmetry systems and two peripheral systems [1–5]. A unit of a symmetry system is composed of a core element at the center and a pair of paracore elements at both sides of a core element [4]. The core element of the border symmetry system is an eyespot, the most conspicuous and most intensively studied element. The prospective eyespot focus at the early pupal stage functions as an organizer for the eyespot color pattern determination as demonstrated by physical damage and transplantation experiments [6–11].

Several candidate genes for eyespot pattern development have been identified based on gene expression studies [9,12–16]. However, morphological studies on the pupal wing tissue and the organizing centers have largely been neglected. We believe that morphological and physiological approaches to the wing system, systematically performed, are necessary to understand the mechanisms of color pattern determination and formation in butterfly wings. For this line of arguments, on the one hand, we have morphometrically examined the scale size, shape, and arrangement of adult wings [17,18]. One of the major findings was the color-size correspondence: scales at the position corresponding to a color pattern element are larger than those of their surroundings [17]. On the other hand, we have developed a method for real-time *in vivo* imaging for pupal wing tissues [17,19–21]. Dynamic pupal epithelial cells were recorded by a real-time confocal fluorescent microscopic technique [19,21]. In addition, we detected spontaneous wing-wide calcium waves and oscillations at the early pupal stage [20]. These studies were mostly performed on the dorsal surface of hindwings of *Junonia orithya*, and we noticed that the pupal wing epithelial area corresponding to the prospective eyespot foci on the dorsal surface of hindwings were resistant to fluorescent staining [19–21], which indicates the three-dimensional structure of the prospective focal area with a thick cuticle layer.

Interestingly, the positions of the prospective eyespot foci on the forewings are identifiable as cuticle spots on the surface of the pupal cuticle [10]. Possible organizers for other color pattern elements are also identifiable as cuticle spots [10]. These pupal cuticle patterns are highly elaborated in nymphalid butterflies, but they can also be seen in other butterflies [10]. Likewise, these pupal cuticle patterns are indicative of the corresponding adult wing color patterns [10]. The pupal cuticle spots for the prospective adult eyespot foci are called focal spots, and they are shown to be correlated with their corresponding adult eyespot sizes in two species of butterflies, which suggests that a focal spot is produced as a direct reflection of the activity of the underlying organizing center [10]. Further characterizations of focal spots and their associated pupal and adult wing tissues might contribute to understand the whole picture of focal spots, eyespot organizers, and eyespots.

In the present study, we examined a size correlation between pupal cuticle focal spots and their corresponding adult eyespots using *Junonia* species. We also examined the three-dimensional surface structure of a focal spot, the underside of a wing pupal case, the pupal wing epithelial tissue underneath a pupal cuticle spot, and adult wing eyespots. We used a high-resolution digital microscope that allowed us to quantitatively examine and reconstruct the three-dimensional structures. The present study is the first characterization of the pupal cuticle focal spots and their associated pupal and adult structures at the microscopic level.

## Materials and Methods

### Ethics statement

The butterflies used in this study were not endangered or protected. No permission is necessary to study these butterflies in Japan.

### Butterflies

Three species of *Junonia* butterflies, *J*. *orithya*, *J*. *almana*, and *J*. *hedonia*, were obtained from the Okinawa-jima and Ishigaki-jima Islands. Females were caught in the field, from which eggs were collected. Larvae were reared on their natural host plants at 27°C under a long-day condition (16L-8D). Adults were frozen after eclosion to avoid physical damage on the wings.

### Images and measurements of pupal focal spots and adult eyespots

We used a Keyence digital microscope VHX-1000 and its associated VHX-2000 communication software version 2.3.5.0. (Osaka, Japan) to take pupal and adult images and to measure the size of the pupal focal spots and adult eyespots using digital images. The same microscope system was used to construct three-dimensional images of the pupal focal spots and adult eyespots.

To measure the pupal focal spot size, one spot was measured 5 times and these raw data were averaged to represent a given focal spot. The height and width data were used to calculate the volume of a spot, assuming that a single spot forms a circular cone. This measurement process was applied to 5 focal spots (from the first to fifth). A set of the spot volume data for these 5 spots from a single pupa was treated as 100% in summation, and percentages of each spot in the summation of 5 volume data were calculated, which yielded the relative focal spot volume. We used 5 pupae per species without sex identification for these measurements and obtained mean and standard deviation values for the relative focal spot volume of a given species. The sixth focal spot was excluded from the measurements because it was difficult to measure due to its small size and high morphological variability. The adult eyespot area was measured in a similar manner; we measured a single sample 5 times using 5 adults per species. We used the ventral black region (inner black disk) at the center of an eyespot. We obtained a set of 5 focal spot data and 5 eyespot data from 2 male and 2 female individuals, and these data were used to construct the scatter plots and to calculate the correlation coefficients. The Shapiro-Wilk normality test indicated that these data were not normally distributed. Thus, we obtained Spearman correlation coefficients and their associated *p*-values.

### Histochemical analysis

A pupal forewing was surgically removed from a pupa 2 days post-pupation together with an associated hindwing and stained with toluidine blue O (Sigma-Aldrich Japan, Tokyo, Japan) as previously described [22]. The tissue was fixed and cryo-protected in phosphate-buffered saline (PBS) containing 4% paraformaldehyde (Kanto Chemical, Tokyo, Japan) and 5% sucrose (Wako Pure Chemical, Osaka, Japan) and stored at 4°C. Then, the portion of the tissue containing the major (fifth) focal spot was cut out, embedded in Tissue-Tek O.T.C. Compound (Sakura Finetek, Torrance, CA, USA), and frozen at -80°C. The tissue block was then serially sectioned at 10 μm and at -30°C using a research cryostat Leica CM1860 (Leica Biosystems, Nusslock, Germany). Bright-field and fluorescent images of the sections were acquired using a Keyence all-in-one fluorescent digital microscope BZ-X710 (Osaka, Japan). For the fluorescent images, a DAPI filter (ex. 360/40; em. 460/50), GFP filter (ex. 470/40; em. 525/50), and TRITC filter (ex. 545/25; em. 605/70) with a metal halide lamp equipped in the BZ-X710 microscope were employed. The cuticle layer thickness and cell layer thickness were digitally measured with the VHX-2000 communication software (Keyence) using these digital images. We obtained serial sets of cuticle layer thickness and underneath cell layer thickness. The Shapiro-Wilk normality test indicated that the thickness data were not normally distributed. Thus, we obtained Spearman correlation coefficients and their associated *p*-values.

## Statistical analysis

Data were compiled and graphically presented with Microsoft Excel. Statistical analyses were performed using the R version 3.0.2 (R Foundation for Statistical Computing, Vienna, Austria).

## Results

### Size of focal spots in *Junonia* species

**Fig 1:**
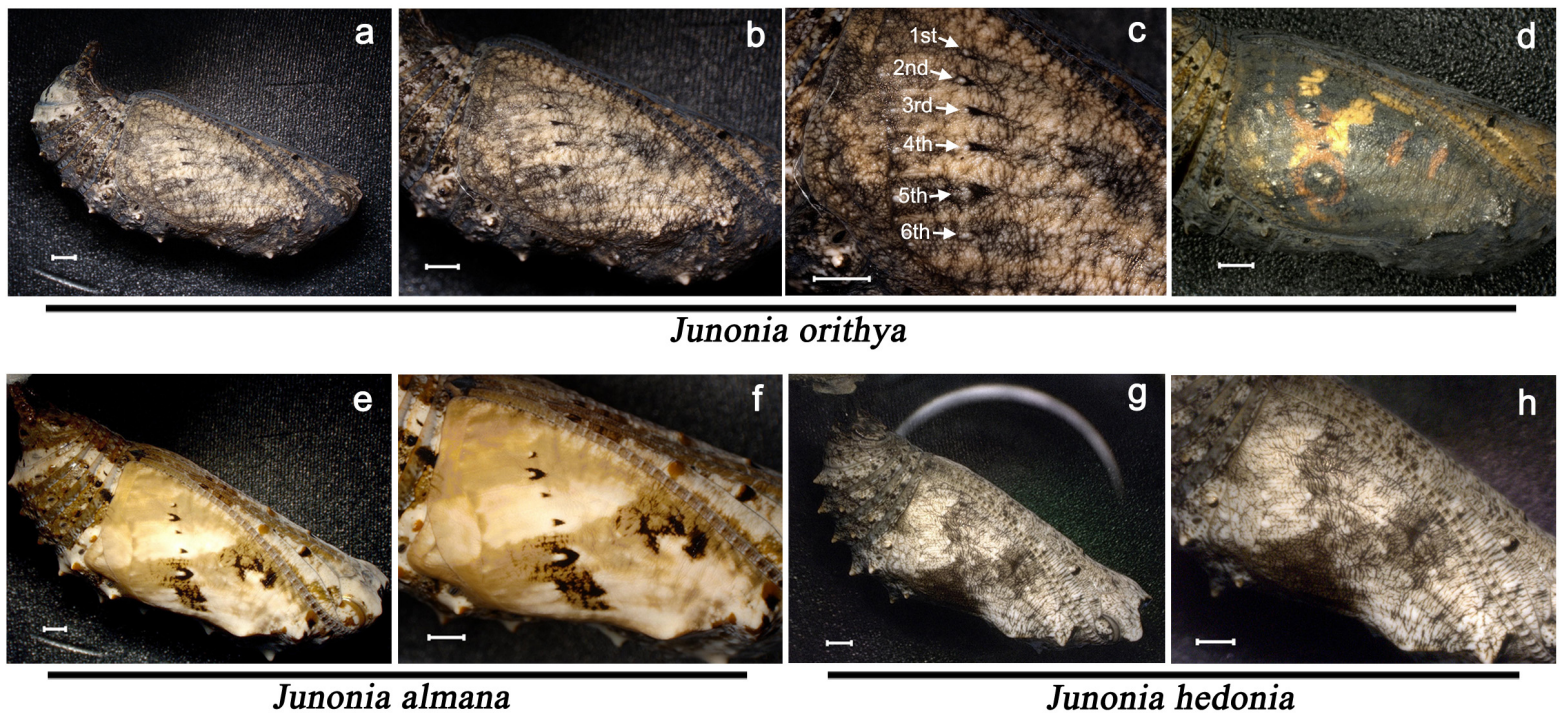
Pupal wing cuticle patterns of 3 *Junonia* species. All scale bars indicate 1 mm. (**a**) A whole *J*. *orithya* pupa. (**b**) A pupal wing surface. High magnification of (a). (**c**) Pupal cuticle focal spots and focal marks (focus-associated marks). They are labelled as first to sixth from the anterior to the posterior sides of a wing. (**d**) A pupal wing surface immediately before eclosion. The adult color pattern is seen through the pupal cuticle case, which demonstrates the correspondence between the pupal focal spots and adult eyespots. (**e**) A whole *J*. *almana* pupa. (**f**) A pupal wing surface. High magnification of (e). Focal spots and marks are observed. (**g**) A whole *J*. *hedonia* pupa. (**h**) A pupal wing surface. High magnification of (g). Focal spots are observed but there is no focal mark.

First, we comparatively examined the wing surface morphology of the pupae of 3 *Junonia* species. They showed unique cuticle patterns (Fig 1). Qualitatively, the large focal spots were found in the species with large eyespots in the adult wings, *J*. *orithya* and *J*. *almana*. Their focal spots were associated with wedge-shaped black cuticle focal marks (or simply, focal marks or focus-associated marks). In contrast, small focal spots were only found in a species with small eyespots in the adult wings, *J*. *hedonia*. Moreover, its focal spots were not associated with cuticle focal marks.

**Fig 2:**
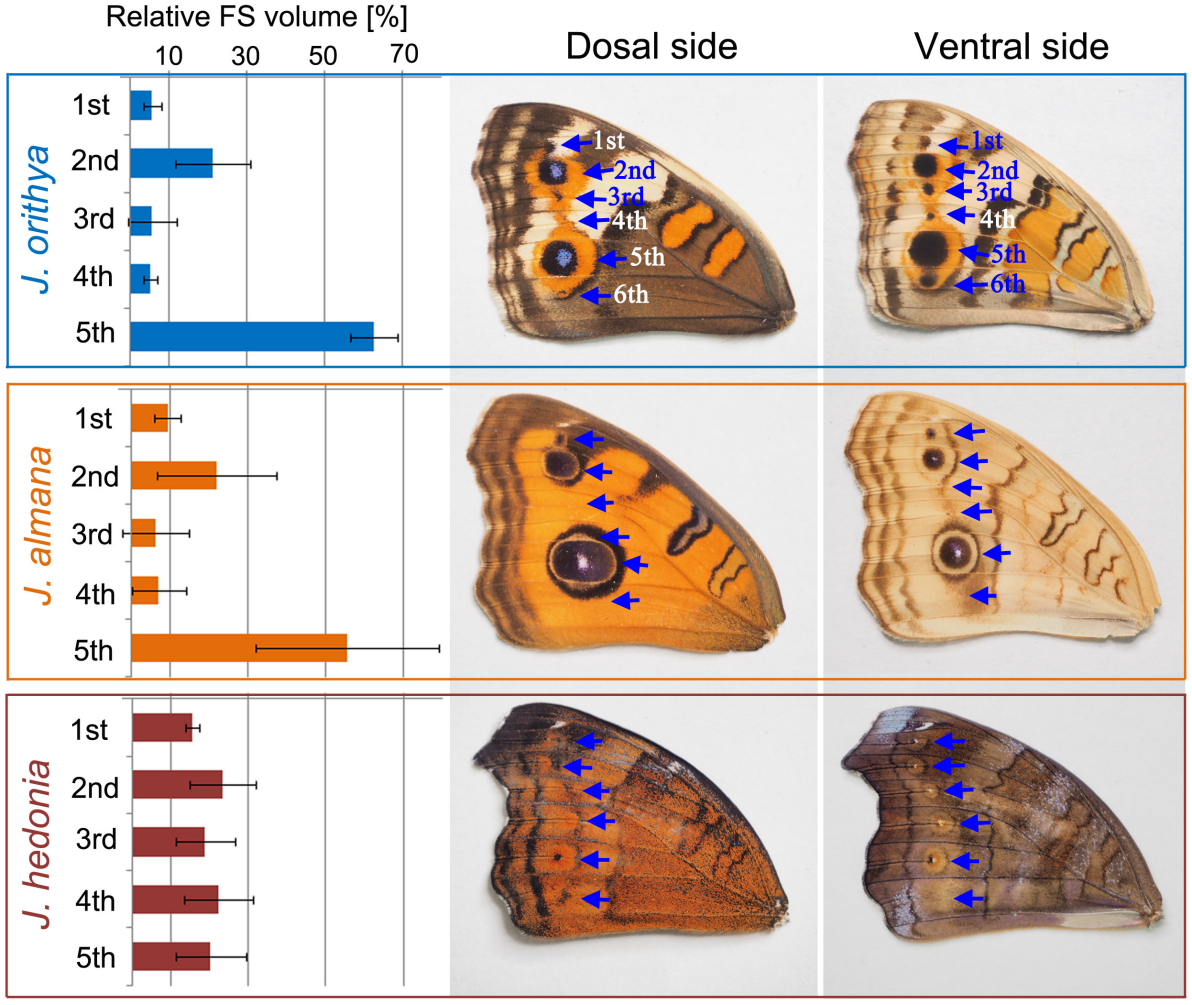
Comparison of the pupal cuticle focal spots and adult eyespots in 3 *Junonia* species. The relative focal spot (FS) volume is shown for 5 focal spots in a species. Mean values are shown and error bars indicate standard deviation. The relative focal spot volume shows that the second and fifth focal spots are larger than the other spots in *J*. *orithya* and *J*. *almana*, whereas all the spots have a similar size in *J*. *hedonia*. A similar pattern is seen in the adult wings of these species. In *J*. *orithya* and *J*. *almana*, the dorsal and ventral eyespot patterns are roughly similar to each other.

The relative size of the 5 focal spots on a single forewing was comparable with the relative size of the corresponding adult eyespots in *J*. *orithya* and *J*. *almana* (Fig 2). More precisely, in these species, the second and fifth eyespots were larger than the others in adult wings, and similarly, the second and fifth focal spots were larger than others in the pupae. In contrast, *J*. *hedonia* has eyespots of similar size in the adult wing and focal spots of similar size in the pupa (Fig 2).

**Fig 3:**
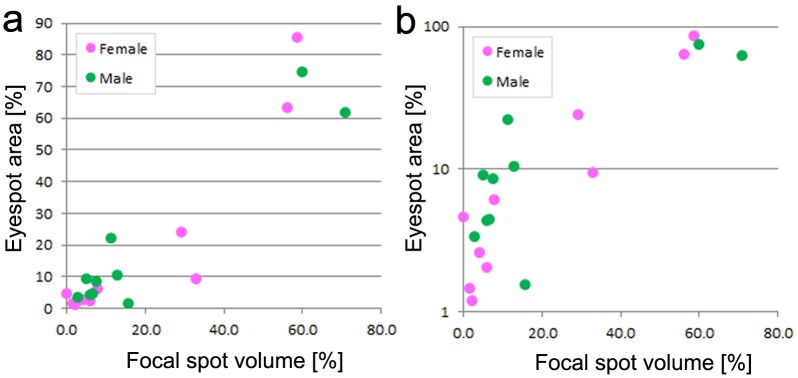
Scatter plots showing the relationship between focal spot volume and eyespot area in *J*. *orithya*. Five focal spots from 2 males and 2 females were examined, showing 20 points in total. Male and female spots are shown in green and pink, respectively. (**a**) Focal spot volume versus eyespot area. (**b**) Focal spot volume versus eyespot area with logarithmic scale.

Quantitatively, the scatter plots between the focal spot volume in the pupae and the eyespot area in adults of *J*. *orithya* (5 focal spot volume data and 5 eyespot area data from 2 males and 2 females) suggested a linear relationship (Fig 3a and 3b). We obtained a Spearman correlation coefficient of 0.788 (*p* = 0.00005).

**Fig 4:**
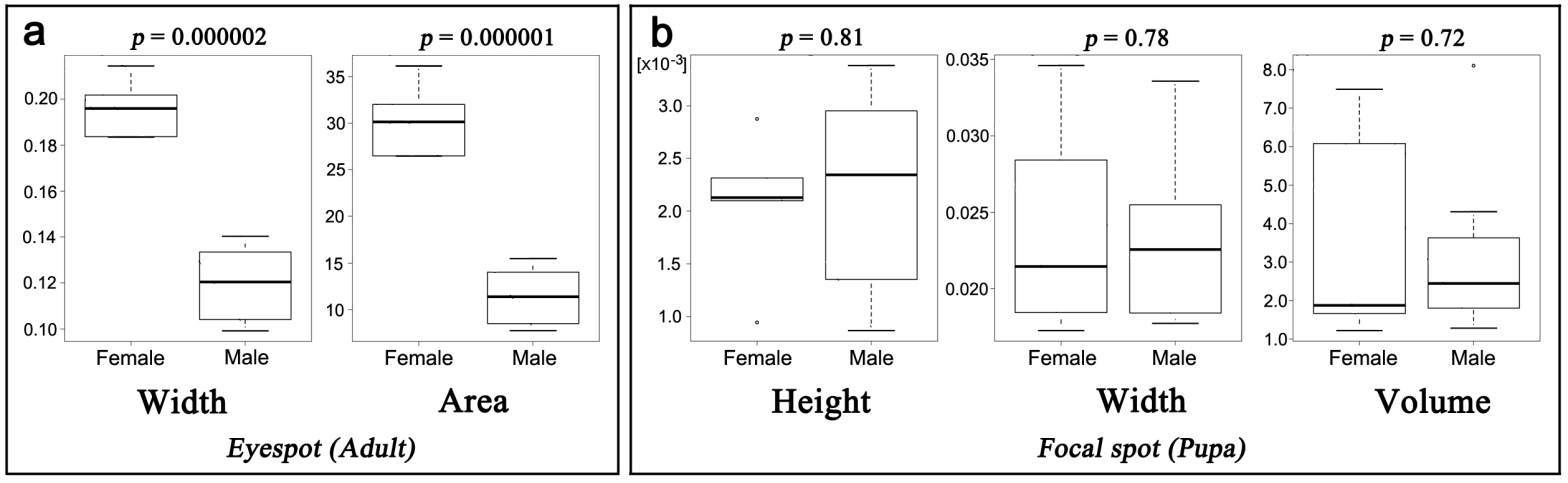
Sexual comparison of the size of adult eyespots and pupal focal spots. (**a**) Adult major (fifth) eyespot. Highly significant differences are detected in width and area. (**b**) Pupal major (fifth) focal spot. No significant difference is detected in height, width, and volume of focal spots between sexes.

A sexual difference in the adult eyespot size was clearly observed in *J*. *orithya* as a dimorphic trait known in this species (Fig 4a). Surprisingly, there were no statistically significant sex differences in height, width, and volume of pupal focal spots (Fig 4b). This result suggest that the level of eyespot inducing activity of the organizing center is similar in both sexes, but other factors affecting the eyespot size, such as hormones, might be different between sexes.

### Fine structures of the *J*. *orithya* pupal surface

**Fig 5:**
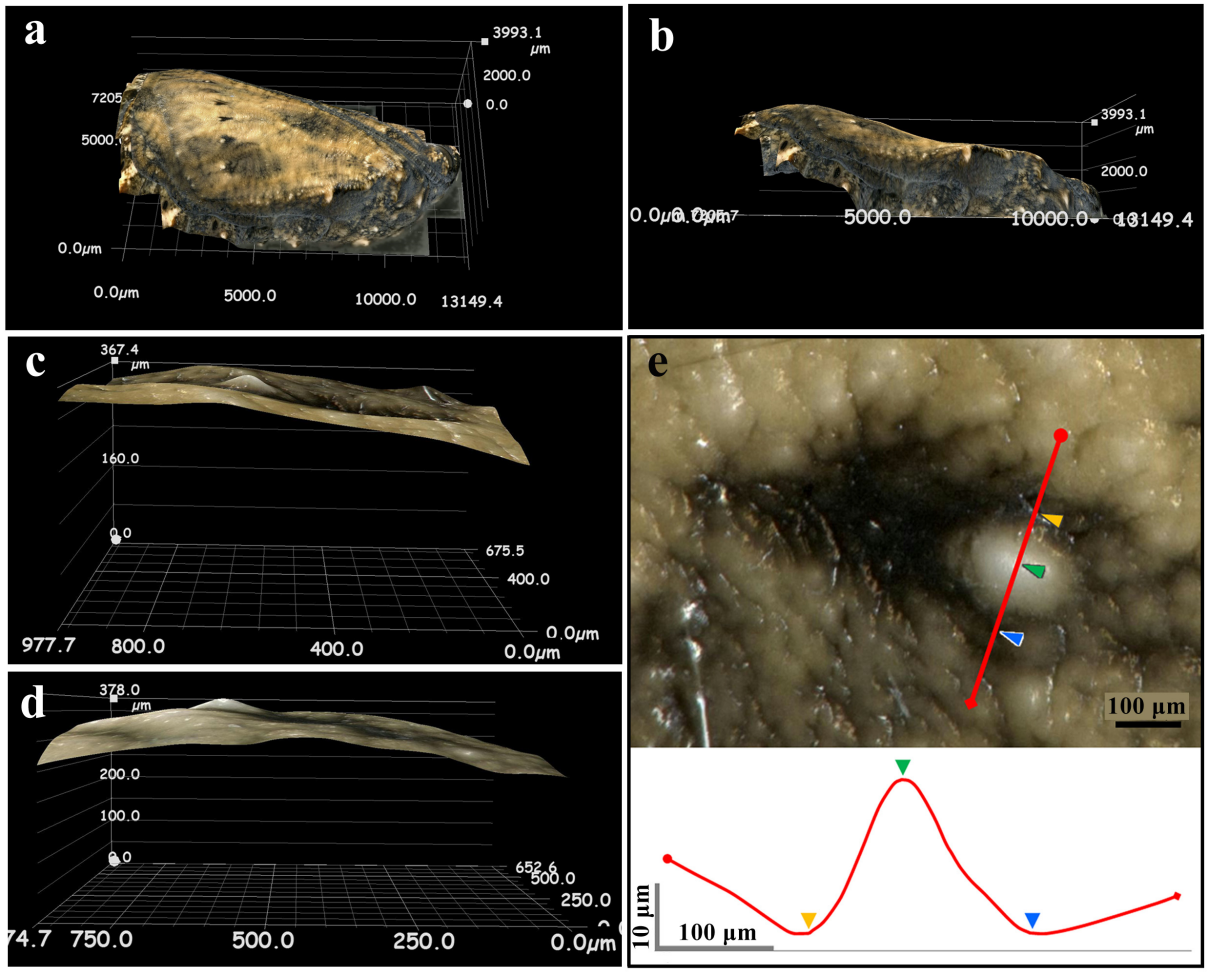
3D structure of the pupal wing surface of *J*. *orithya*. **(a)** A whole pupal wing surface. **(b)** A side view of a pupal wing surface. **(c)** A side view of a region of the major (fifth) focal spot. **(d)** Another side view of a region of the major (fifth) focal spot. **(e)** Size measurement of a focal spot. Diameter and height are approximately 200 μm and 20 μm, respectively. The black region is a focal mark (focus-associated mark), which is lower in height than its surroundings. Red lines and colored arrowheads in top and bottom panels indicate identical sites.

Hereafter, we mainly focused on *J*. *orithya*. The pupal surface was three-dimensionally reconstructed using a digital microscope (Fig 5a–5d). The fifth focal spot was clearly seen as a bulge on the surface. Detailed size measurements also confirmed this bulge structure (Fig 5e). The spot exhibited a gentle cone shape with a height of more than 20 μm and a bottom diameter of approximately 200 μm. The region of the black focal mark was found to be sunken below its surroundings.

**Fig 6:**
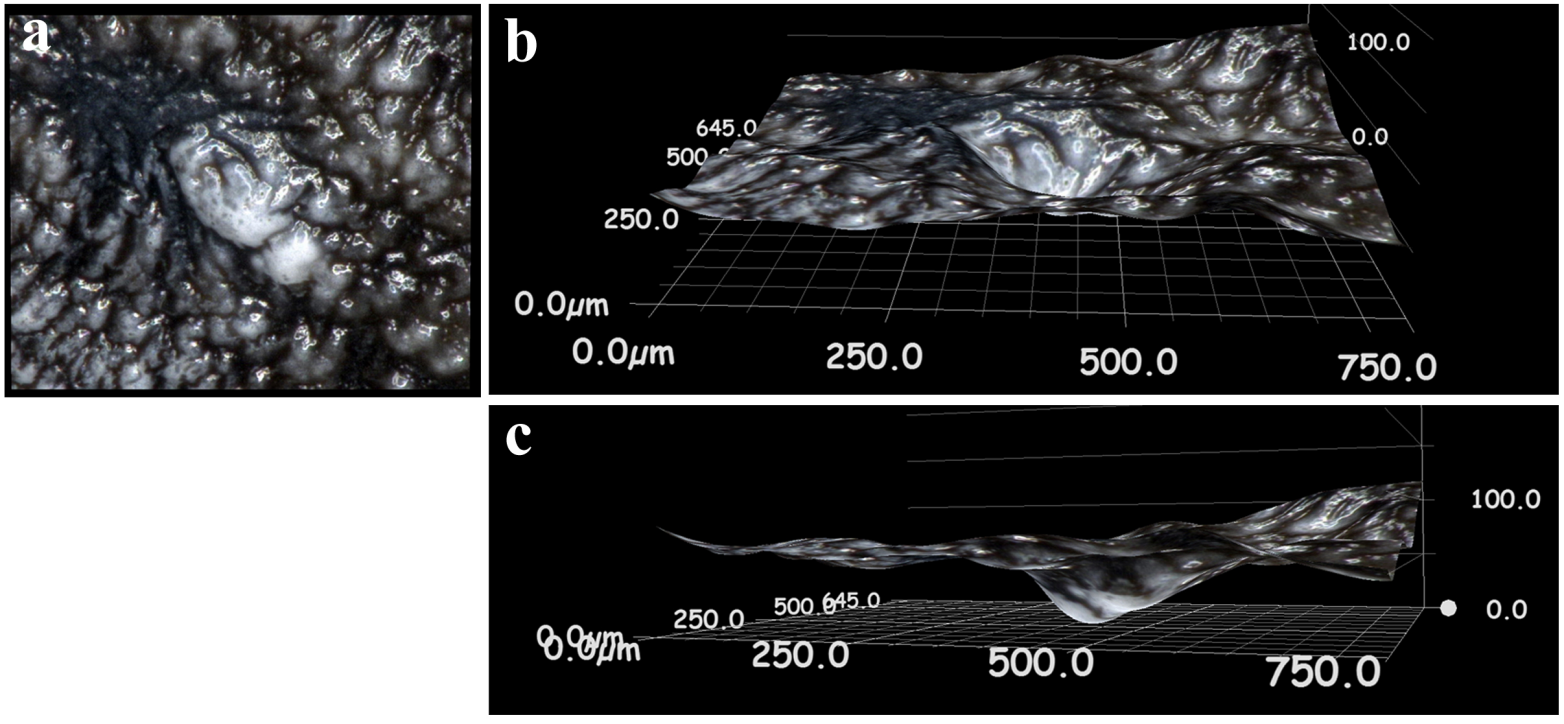
3D structure of the underside of a *J*. *orithya* pupal case at a focal spot. **(a)** An image of a pupal case upside down at a focal spot. **(b)** An obliquely positioned view. **(c)** A side view.

We also examined the underside of a pupal case after eclosion (post-eclosion pupal shell) in 3D images. The underside of the major (fifth) focal spot exhibited a hollow inside (Fig 6). This observation raised the possibility that a focal spot is produced simply by a curved cuticle. Alternatively, but not mutually exclusively, a focal spot might be produced by a thickened cuticle at that site.

**Fig 7:**
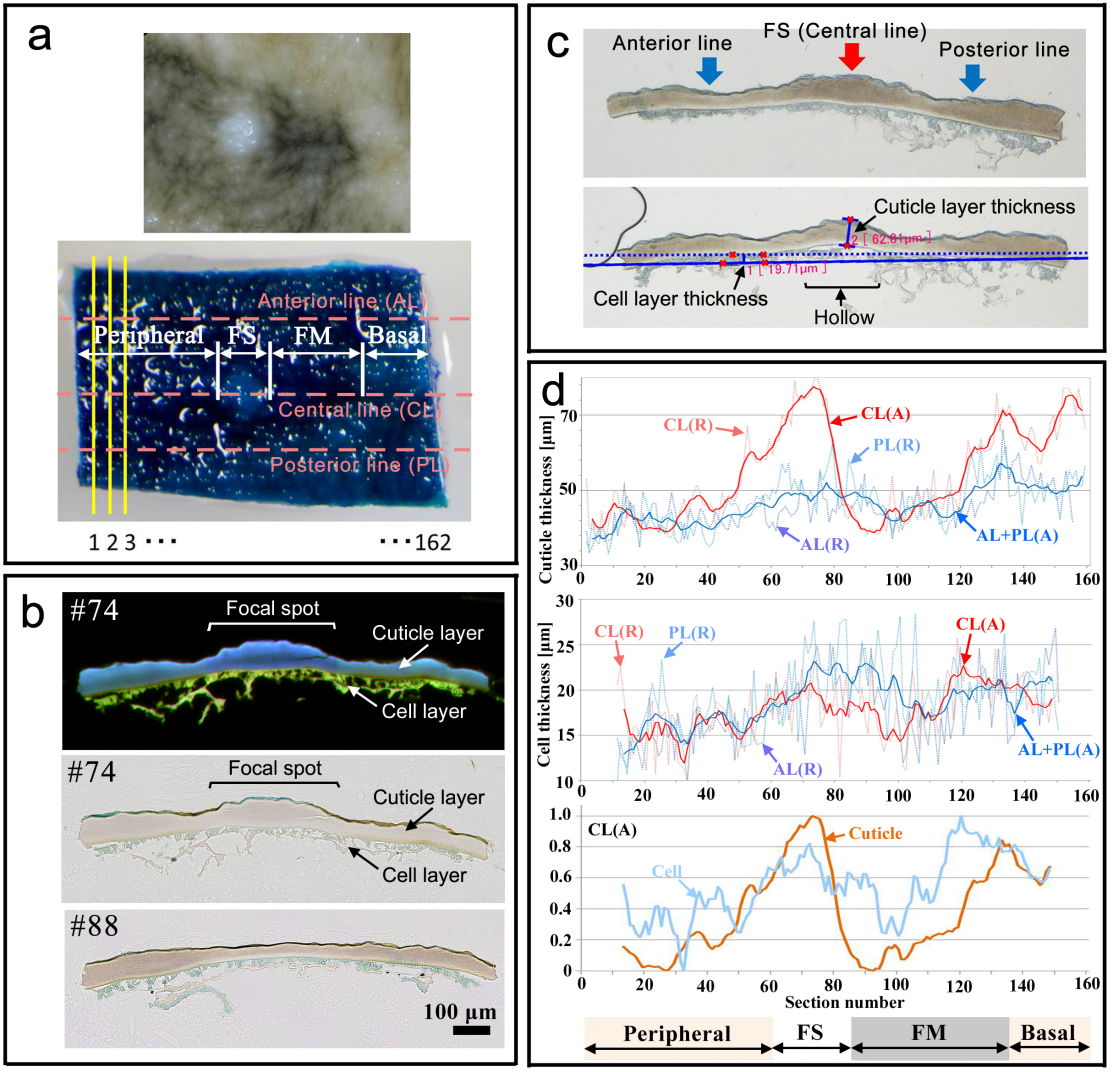
Cross section of the pupal major (fifth) focal spot of *J*. *orithya*. **(a)** An excised region of a focal spot. The non-stained image is at the top, and the toluidine blue-stained image is at the bottom. Sections were made along yellow lines from peripheral to basal regions. Section numbers are shown below the image. The epicuticle is stained by toluidine blue. Four regions are defined: focal spot (FS), focal mark (FM), and basal and peripheral regions. The thickness of the cuticle layer and the underneath cell layer were measured along the anterior, central, and posterior lines shown in pink. (**b**) Examples of sections. The autofluorescent image (top) is a combination of three (blue, green, and red) fluorescent images. A blue signal indicates the cuticle, whereas a green signal indicates the cell layer (wing tissue). In the two bright-field images (middle and bottom), the light brown layer is the pupal cuticle, and the light blue layer below is the cell layer (wing tissue). The epicuticle is also stained with blue on the surface of the cuticle. The section number is indicated at the top left-hand corner. (**c**) Three measurement points (anterior, central, and posterior) (top) and how to measure the cuticle layer thickness and cell layer thickness (bottom) using a VHX-2000 communication software (Keyence). A hollow is seen underneath the focal spot. (**d**) Cuticle thickness (top panel) and cell thickness (middle panel) along the central, anterior, and posterior lines. Raw data are plotted with dotted lines, and averaged data over 5 sections (the section at a given point and two sections before and after that point) are plotted with solid lines. The averaged data for the cuticle layer and cell layer along the central line are shown as relative values (a maximum point is adjusted to be 1.0; bottom panel), indicating a positional correspondence between the cuticle thickness and cell thickness. Below the three panels, the 4 regions of the sections are indicated.

To examine these possibilities, the major (fifth) focal spot together with its associated pupal wing tissue was isolated and subjected to histochemical analysis (Fig 7a). We made cross sections of a piece of cuticle with wing tissue underneath after staining it with toluidine blue (Fig 7b). Toluidine blue stained the cuticle surface (i.e., epicuticle) and the cell layer below the cuticle layer (Fig 7b). The cuticle and cell layers were easily distinguishable by autofluorescence; the cuticle layer (but not cell layer) exhibited blue fluorescence under the excitation of ultraviolet light (Fig 7b).

**Fig 8:**
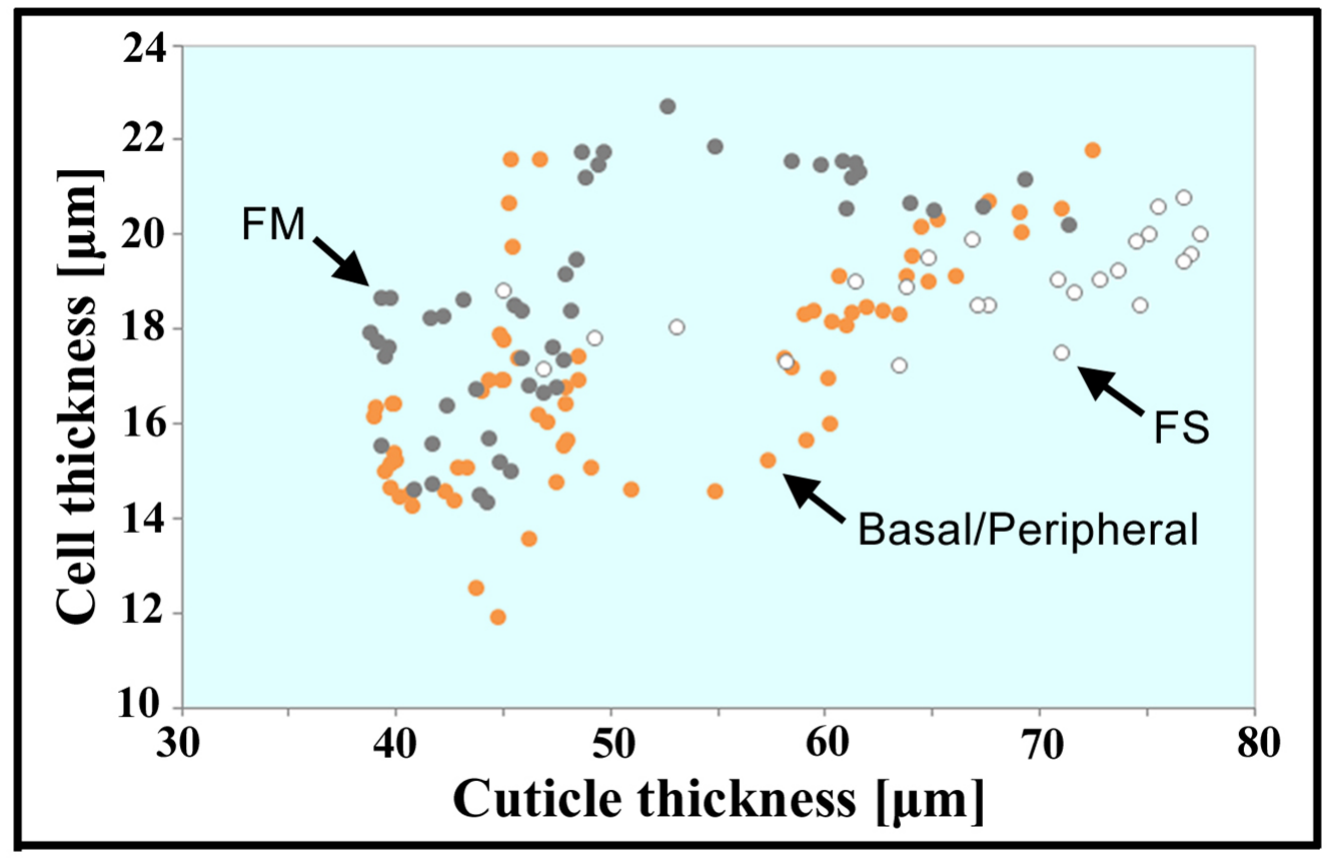
Scatter plot of the relationship between the cuticle thickness and cell thickness. Three regions, i.e., focal spot (FS), focal mark (FM), and basal/peripheral regions, are indicated in different colors.

Qualitatively, the cuticle layer of a pupal focal spot was thicker than its surroundings and showed a curvature; the focal spot had a hollow underside (Fig 7b and 7c). Quantitatively, we measured the thickness of the cuticle and the thickness of the underneath cell layer along the anterior, central, and posterior lines at each section (Fig 7a and 7c). At the focal spot, both the cuticle and cell layers showed a peak of thickness along the central line (Fig 7d). The cuticle layer of the focal spot was as thick as 80 μm at its maximum, whereas the cuticle layer of the non-focal spot regions along the anterior and posterior lines was 40–60 μm (Fig 7d, top). The thickness of the cell layer showed a modest peak at the position of the focal spot, which was approximately 20 μm (Fig 7d, middle). The peaks of the cuticle and cell layer thickness along the central line extensively coincided (Fig 7d, bottom). The scatter plots between the cell layer thickness and the cuticle layer thickness suggested a weak linear relationship between them (Fig 8). A size correlation was obtained for the entire tissue region sectioned (*ρ* = 0.44; *p* = 0.0000001; *n* = 130 sections) and in 3 separate regions, i.e., the focal spot region (*ρ* = 0.36; *p* = 0.08; *n* = 25 sections), the focal mark region (*ρ* = 0.46; *p* = 0.001; *n* = 47 sections), and other (basal and peripheral) regions (*ρ* = 0.48; *p* = 0.0001; *n* = 58 sections). These results suggest a reasonable correlation between the cuticle layer thickness and the cell layer thickness.

### Adult eyespot structures

**Fig 9:**
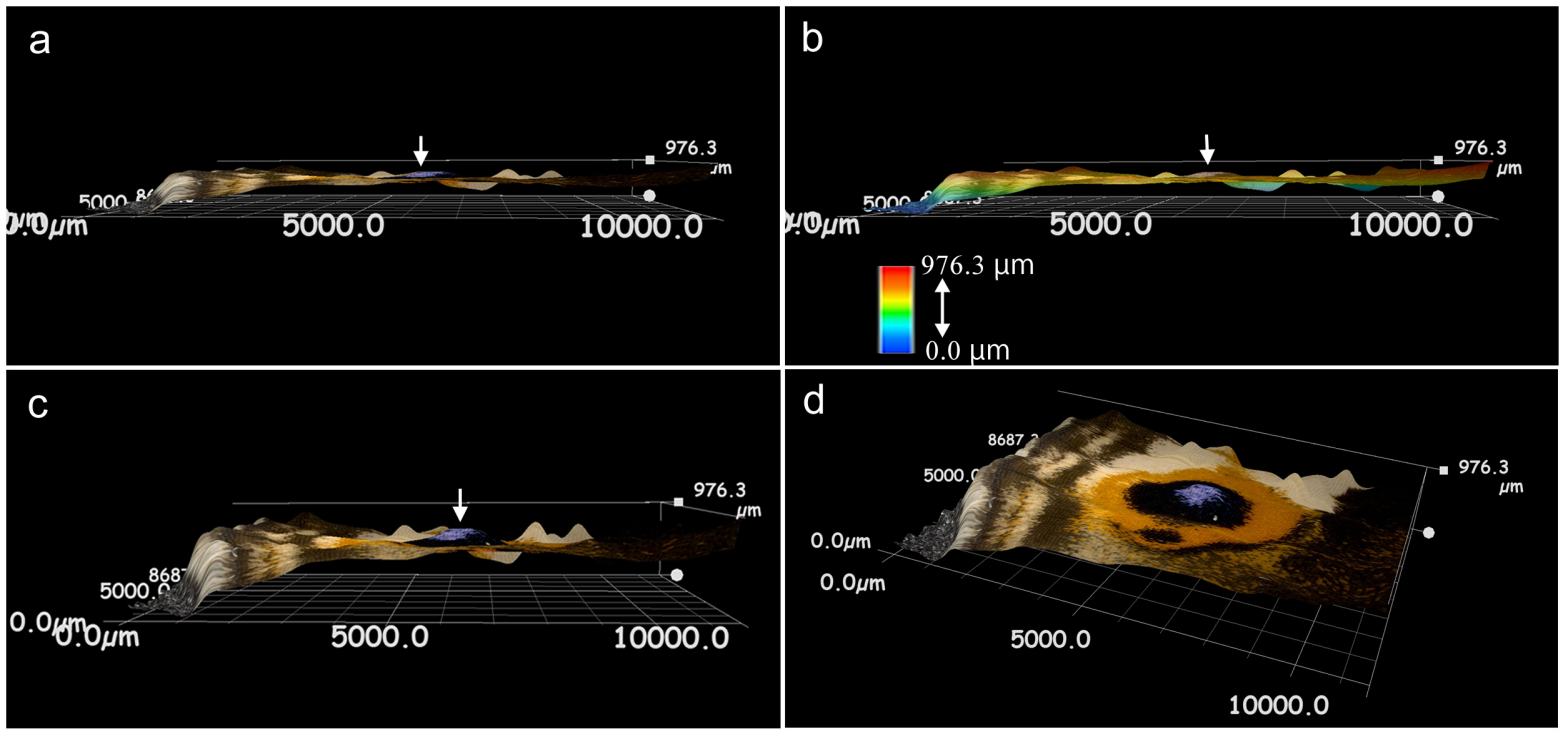
3D structure of the eyespot of a *J*. *orithya* adult female. Arrows indicate an eyespot focus. (**a**) A side view. (**b**) A side view with color scale. (**c**) Side view with expanded height scale (× 2). (**d**) An obliquely positioned view with expanded height scale (× 2).

The bulge of the cell layer of the future wing suggests that the adult wings might also have a similar bulge. As expected, we found that the focus of the adult eyespot exhibited a surface elevation, as shown in the 3D wing images of the *J*. *orithya* female major (fifth) eyespot (Fig 9).

**Fig 10:**
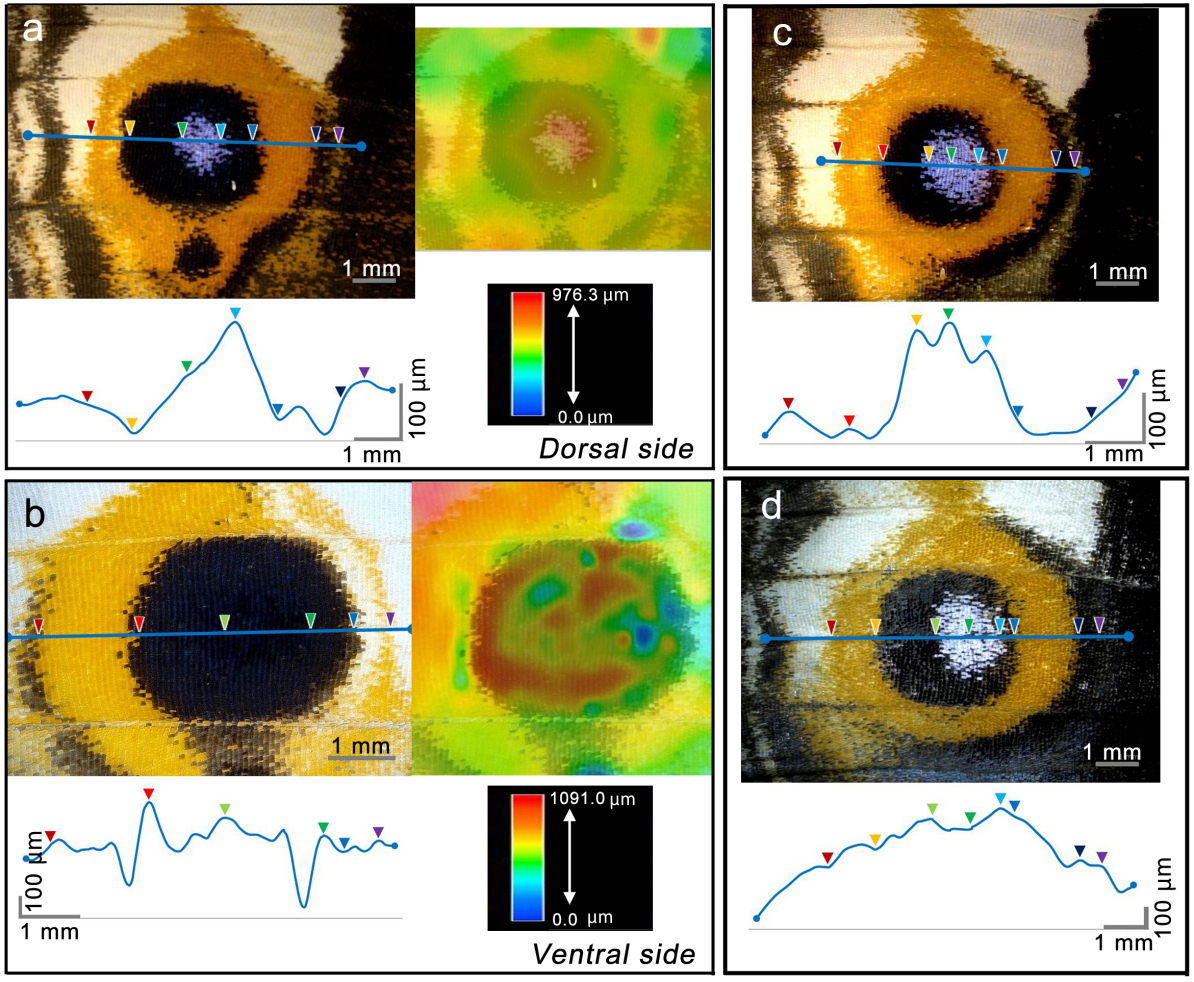
Height measurements of the eyespots of a *J*. *orithya* adult female. Blue lines and colored arrowheads indicate identical sites in the top and bottom images of each panel. (**a**) A dorsal eyespot with color scale. (**b**) A ventral eyespot with color scale. Eyespots shown in (a) and (b) are on the opposite surface on the same wing. (**c**, **d**) Additional dorsal eyespots from 2 individuals.

A further quantitative analysis confirmed that a height peak corresponded to a blue focal area of a dorsal eyespot in *J*. *orithya* females (Fig 10a, 10c and 10d). From the focal peak to the yellow ring through the black inner disk, the height decreased steeply in 2 individuals (Fig 10a and 10c). In these 2 individuals, the height difference from the peak to the bottom was approximately 200 μm (Fig 10a and 10c). In the third individual, we found a different height pattern (Fig 10d). A clear focal peak was not found in the ventral eyespot (Fig 10b). This is probably because the ventral eyespot does not have a focal area (Fig 10b). It appeared that the outer black ring (expressed only at the proximal side) adjacent to the yellow ring also exhibited a small level of surface elevation, but the yellow ring was not elevated. Hence, there was a color-height correspondence within an eyespot.

**Fig 11:**
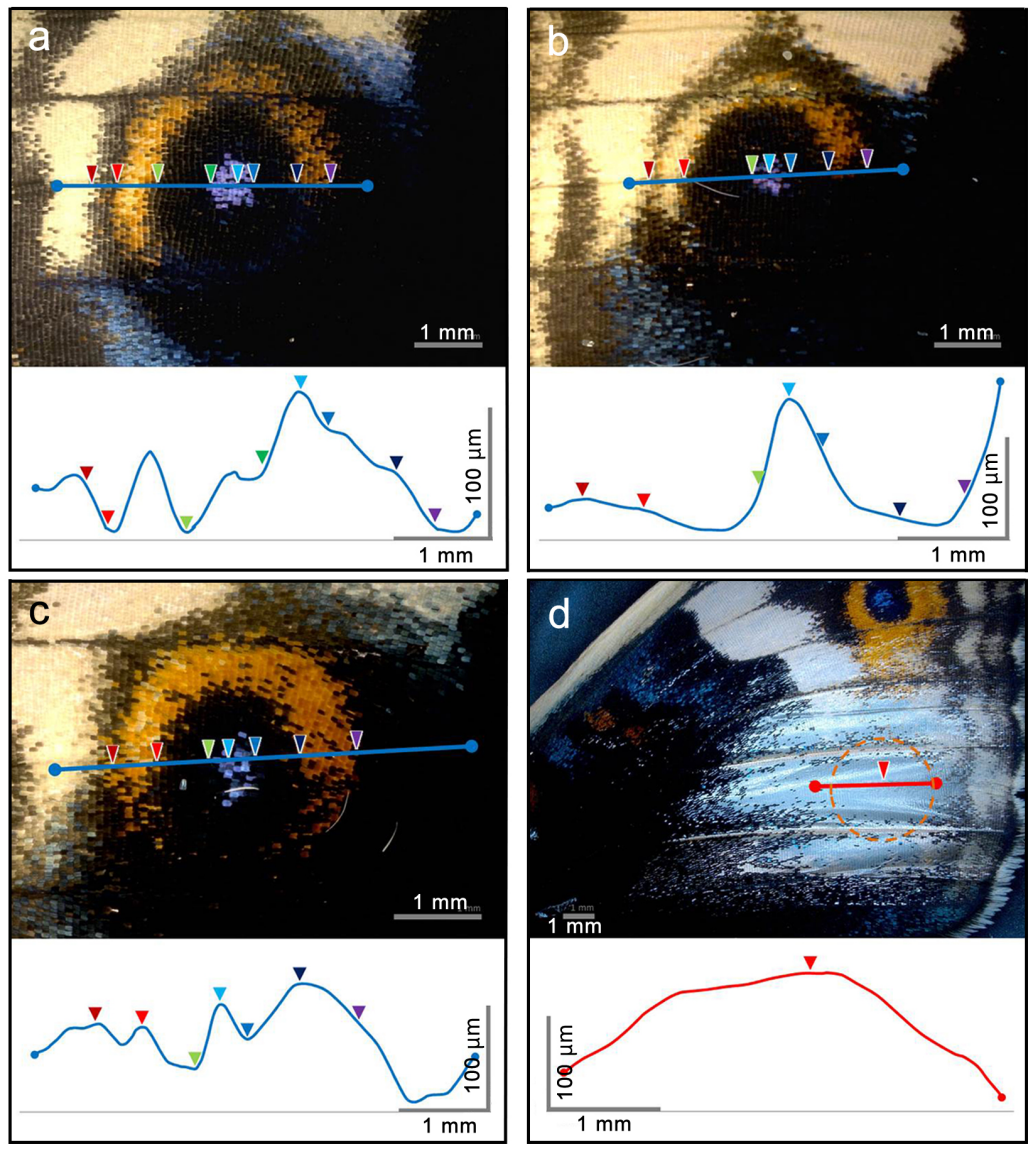
Height measurements of the dorsal eyespots of a *J*. *orithya* male. Blue lines and colored arrowheads indicate identical sites in the top and bottom images of each panel. (**a-c**) Eyespots from 3 different individuals. (**d**) Eyespot region with scales removed. The eyespot region is circled.

We also examined the dorsal major (fifth) eyespot of *J*. *orithya* males and obtained similar results (Fig 11a–11c). Interestingly, the level of surface elevation at the blue focal region was approximately 100 μm from the level of the yellow ring, which was approximately half of the female value. The removal of scales from the wing surface did not eliminate this bulge structure (Fig 11d). The sharpness of a peak appeared to be lost in the process of scale removal due to the physical damage produced during the removal process and to the transparency of the sample, which likely made the optical height measurement difficult. Regardless of these complexities, our data demonstrated that the wing basal membrane was three-dimensionally constructed.

**Fig 12:**
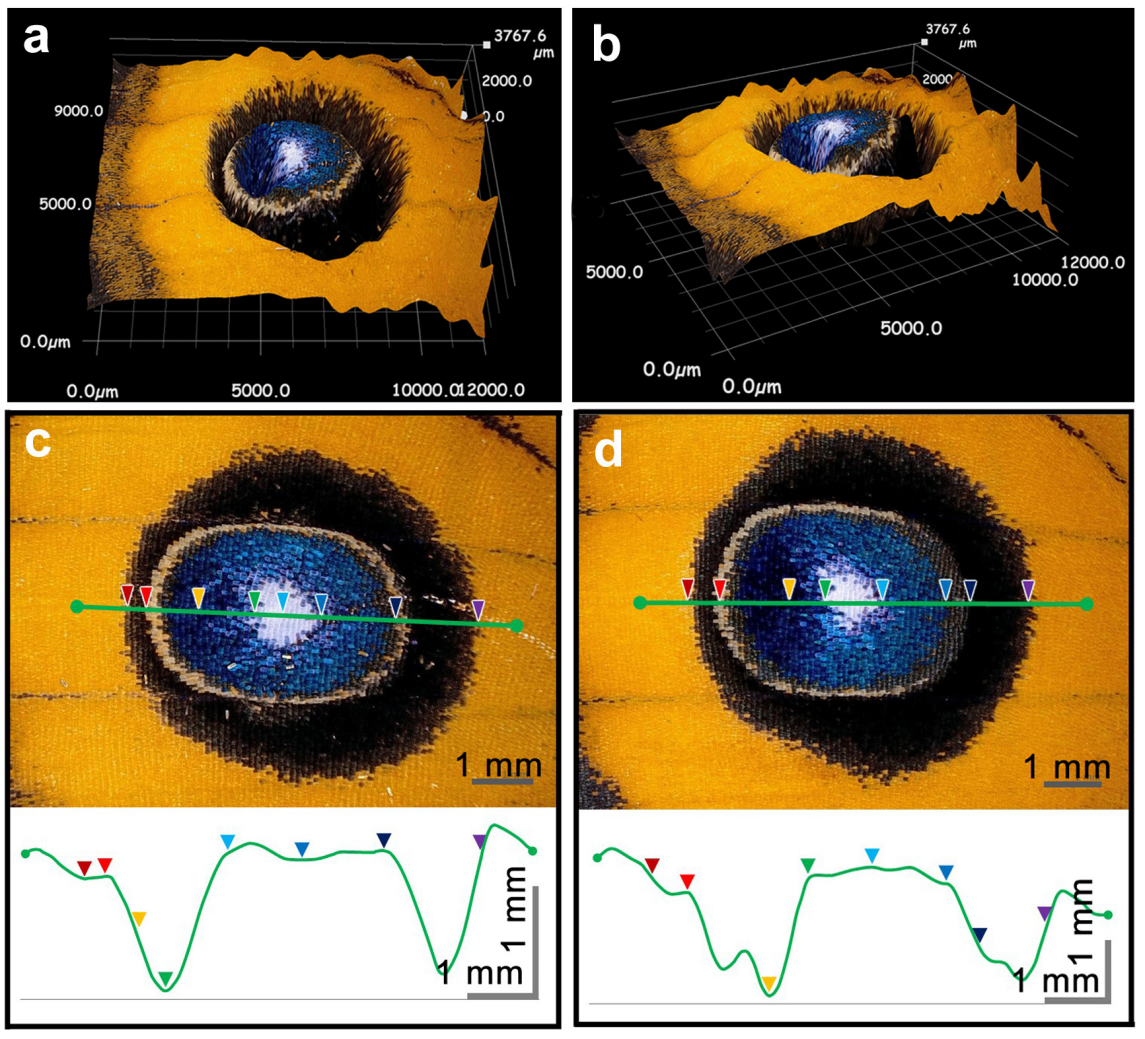
3D structure and height measurements of the dorsal eyespots of *J*. *almana*. (**a**) A top-down view of the 3D structure. (**b**) An obliquely positioned view of the 3D structure. (**c, d**) Eyespots from 2 different individuals. Green lines and colored arrowheads indicate identical sites in the top and bottom images of each panel.

Similar analysis using *J*. *almana* revealed a clearer color-height relationship; the 3D reconstruction of an eyespot visually indicated that the height approximately corresponded to the eyespot coloration (Fig 12a and 12b). Quantitative height analysis showed that the focal area and its associated blue structural color area formed a high flat basin, and the black regions were depressed (Fig 12c and 12d), which indicates a reasonable color-height correspondence. The height difference (not to be confused with wing thickness) was more than 1 mm (*n* = 2).

## Discussion

In this study, we morphometrically investigated the pupal focal spots and their associated pupal and adult wing structures as a continuation of a previous study [10]. The pupal focal spots are physically located immediately above the prospective eyespot focus [10]. Thus, their morphological and physiological relationship is of great interest. In *J*. *orithya*, a late larval wing tissue does not clearly have a specific structure at the prospective eyespot focal area [21], whereas an early pupa immediately after pupation has focal spots [10]. Therefore, the focal spots and their associated structures are probably produced at the prepupal stage.

As expected, a large eyespot is likely accompanied by a large pupal focal spot in *J*. *orithya* and *J*. *almana*. This cross-species comparison clearly reflects the facts that the two *Junonia* species that have large eyespots (i.e., *J*. *orithya* and *J*. *almana*) have large pupal focal spots and that the *Junonia* species that has small eyespots (i.e., *J*. *hedonia*) has small pupal focal spots. Quantitatively, the eyespot area in the adults was correlated with the focal spot volume in the pupae. Probably, the size of the pupal focal spots is a reflection of the organizing activity of the pupal wing epithelial cells underneath the focal spot. In addition, focal marks are found in *J*. *orithya* and *J*. *almana* but not in *J*. *hedonia*. Focal marks are also likely associated with the activity of the prospective eyespot cells underneath the cuticle.

However, we were unable to obtain statistically significant differences of the pupal major (fifth) focal spot in size between sexes in *J*. *orithya* despite the clear sexual difference in the adult major (fifth) eyespot. This result would not overturn the proposed physiological association between the pupal focal spot and the underneath eyespot organizer. Instead, this result might suggest that the levels of activity of the organizing centers are similar between sexes, but there are other sexually different factors that modify the final eyespot size, such as the ecdysteroid and cold-shock hormone in the hemolymph.

The underside of the pupal cuticle immediately below a focal spot using a post-eclosion pupal case revealed a hollow underneath a focal spot. Cross sections of the pupal wing cuticle and tissue revealed a curvature of the cuticle to make a hollow, which is consistent with the observation of a post-eclosion pupal case. The pupal wing epithelial tissue (i.e., the focal organizing center) probably forms a non-flat structure that fits into that space. Quantitatively, both the focal spot cuticle layer and the cell layer underneath the focal spot were thicker than their surrounding areas; the thickness of these layers was correlated. Therefore, it is likely that the curvature and thick cuticle were produced by the activity of large cells underneath the focal spot.

Considering these facts, it might not be surprising to discover that the adult wing eyespots are not flat but three-dimensional. Although height patterns varied among individuals and between the two species examined, the focal area and its associated area with structural colors usually showed an elevation, and the adjacent black area showed a depression. Non-black rings usually showed another elevation. This discovery of the color-height relationship might have an implication in a fate determination mechanism of immature scale cells. However, it is true that the adult height and its pattern vary considerably and the exact quantitative definition of height is difficult in wing samples. Moreover, precise quantitative values may depend on the digital microscope used in this study. Further examination with different measurement methods may be necessary for quantitative discussions. In this sense, the possible sex difference in height in *J*. *orithya* adult wings, which is surprising considering that there was no sex difference in height in the pupal focal spots, awaits verification.

We have previously shown that the eyespot color patterns of actual butterflies from various species are highly complex and that they cannot be thoroughly explained by the classical morphogen gradient models [23]. We have proposed an alternative model called the induction model, in which a train of wave pulses from the focus act as morphogenic signals [23–25]. These signals dynamically induce inhibitory signals around themselves, but they can also induce a secondary organizing center [24,25]. This model is applicable to the eyespot behaviors after physical damage [11] and chemical modifications [26]. Likewise, the induction model is applicable to the black spot behavior of genetic mutants [27]. The physical curvature or distortion of the pupal wing epithelial tissue, which might be introduced by a physical structure of the cuticle and be similar to a physical damage, might play an important role in a signaling process that is explained by the induction model.

## Conclusions

Pupal cuticle focal spots are correlated with adult eyespots in size and exhibit a surface elevation and a curvature of the cuticle layer itself. The cell layer underneath the cuticle layer at the pupal cuticle focal spots also shows a curvature. These two layers are correlated with each other in thickness. Probably because of these structural features, adult eyespots are three-dimensionally constructed. The color-height relationship in adult eyespots may reflect a developmental mechanism for eyespot determination in butterflies.
